# Dark Future: Development and Initial Validation of Artificial Intelligence Conspiracy Beliefs Scale (AICBS)

**DOI:** 10.1002/brb3.70648

**Published:** 2025-07-03

**Authors:** Chung‐Ying Lin, Julia Brailovskaia, Servet Üztemur, Ali Gökalp, Nail Değirmenci, Po‐Ching Huang, I‐Hua Chen, Mark D. Griffiths, Amir H. Pakpour

**Affiliations:** ^1^ Institute of Allied Health Sciences, College of Medicine National Cheng Kung University Tainan Taiwan; ^2^ Department of Public Health, College of Medicine National Cheng Kung University Tainan Taiwan; ^3^ Biostatistics Consulting Center, National Cheng Kung University Hospital, College of Medicine National Cheng Kung University Tainan Taiwan; ^4^ School of Nursing, College of Nursing Kaohsiung Medical University Kaohsiung Taiwan; ^5^ Mental Health Research and Treatment Center Ruhr‐Universität Bochum Bochum Germany; ^6^ German Center for Mental Health (DZPG) partner site Bochum‐Marburg Bochum Germany; ^7^ Department of Turkish and Social Sciences Education, Faculty of Education Anadolu University Eskişehir Türkiye; ^8^ Department of Educational Sciences Gaziantep University Gaziantep Türkiye; ^9^ Institute of Educational Sciences Gazi University Ankara Türkiye; ^10^ Department of Physiotherapy, School of Nursing and Health Sciences Hong Kong Metropolitan University Hong Kong China; ^11^ Chinese Academy of Education Big Data, Faculty of Education Qufu Normal University Qufu China; ^12^ International Gaming Research Unit, Psychology Department Nottingham Trent University Nottingham UK; ^13^ Department of Nursing, School of Health and Welfare Jönköping University, Hälsohögskolan Jönköping Sweden

**Keywords:** artificial intelligence, artificial intelligence conspiracy theories, conspiracy theories, generative artificial intelligence, psychometric testing, scale development

## Abstract

**Background:**

In the past few years, the rapid development of artificial intelligence (AI) and its success in many areas of everyday life have attracted global attention. Some discussions have noted that generative AI tools can make decisions on their own with the potential to improve themselves. Subsequently, conspiracy theories have emerged concerning the future implications of AI. In the present study, the Artificial Intelligence Conspiracy Beliefs Scale (AICBS) was developed to assess conspiracy beliefs concerning AI, andits psychometric properties were examined.

**Methods:**

A cross‐sectional survey was conducted with 788 Turkish participants (*M*_age _= 25.10 years, 56% female). The sample was split to carry out an exploratory factor analysis (EFA; *n *= 423) and a confirmatory factor analysis (CFA; *n *= 365), resulting in a 30‐item scale comprising five subdimensions.

**Results:**

The five‐factor structure explained 62.58% of the total variance. The CFA showed acceptable model fit indices and confirmed the EFA's five‐factor structure. Based on the EFA's factor loadings, a short five‐item version of the AICBS (AICBS‐5) was developed with one item from each subdimension (which explained 45.28% of the variance). The CFA confirmed the unidimensional structure of the AICBS‐5. The internal consistency coefficients of the AICBS, its subdimensions, and the AICBS‐5 demonstrated very good reliability. Correlation analyses with external criterion measures (AI Anxiety Scale, Generic Conspiracist Beliefs Scale‐5, and Anomie) supported the concurrent validity of the AICBS, its subdimensions, and the AICBS‐5.

**Conclusion:**

The findings demonstrate that both AICBS and AICBS‐5 are valid and reliable psychometric instruments to assess AI conspiracy beliefs.

## Graphical abstract

The Artificial Intelligence Conspiracy Beliefs Scale (AICBS) was developed and validated using data from 788 participants to assess conspiracy beliefs about artificial intelligence. EFA resulted in a five‐factor structure (Interpersonal Relations and Social Influence, Arms Rivalry and Less World Peace, Human Labor and Human Intelligence, Global Control, and Disinformation), explaining 62.58% of the variance, which CFA confirmed. Both the full scale and the short form (AICBS‐5) demonstrated good validity and reliability.

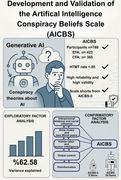

## 1 Introduction

The rapid development and integration of artificial intelligence (AI) in different areas of human life over the past few years have increased efficiency and productivity (Sowa et al. 2021; Tasheva and Karpovich 2024), while working costs have significantly decreased (Liu and Li 2024). The effective use of AI can make daily routines easier, more efficient, and more useful than in the past. It has also paved the way for innovative solutions that benefit individuals in their daily work and has facilitated the usability of big data (Zhang 2023). The contribution of AI to society and its wide range of applications has led to rapid and radical developments (Yang 2022). AI has penetrated humanity rapidly and has had an intense, transformative effect on both individuals and society.

Although AI has brought many benefits (e.g., Wu and Zhang 2022), it has also become the focus of dark conspiracy theories. Since the concept of AI was first introduced seven decades ago (McCarthy et al. 2006), the rapid pace of developments in this field has led some to worry about the future impact of AI. Artificial Narrow Intelligence (ANI) refers to AI systems designed for specific tasks and has achieved significant success in machine learning (Shadbolt 2022). However, its ability to infer or generalize is limited. It has not been viewed as a worry because it cannot go beyond its own narrow framework of understanding and coded responses (Kuusi and Heinonen 2022). Artificial General Intelligence (AGI) refers to machine intelligence that can perform any intellectual task that a human can perform (Chehreghani 2024). Some researchers argue that AGI is unattainable (Fjelland 2020), while others argue that it is achievable (Chehreghani 2024; Mitchell 2024). Moreover, the potential of AGI to outperform human intelligence in cognitive tasks such as problem‐solving and adaptation has been emphasized (Groppe and Jain 2024; McLean et al. 2021).

Another type of AI, Artificial Super Intelligence (ASI), is often referred to as a type of AI substantially higher than human intelligence. It can quickly solve complicated problems, self‐educate, and learn (Novikov 2024). The rapid integration of AI technologies, which are rapidly advancing towards becoming AGI and ASI, into critical areas (e.g., cybersecurity, health, and education) has brought along threats to professional identity, transformative impact on the labor force, and ethical concerns (e.g., cashiers and translators could be replaced by AI) (Jussupow et al. 2022; Richie 2022; Wang 2024). Moreover, in the absence of controllability and transparency, it is foreseen that uncertainties and misinformation will continue to increase in society against AI. Consequently, it is thought that motion pictures, science fiction‐based printed works, and mythology about AI will feed the concerns on this issue and cause it to become a conspiracy theory (Carillo 2020; Gherkeş 2018).

Despite its many positive aspects, the probability that AI can develop in ways that may exceed human capacity causes anxiety and fear among many individuals (Gherheş 2018). This may lead to conspiracy theories about AI. These concerns often stem from unpredictability and unknowability. Concerns that it will take away people's jobs, turn into a mass weapon under the control of dangerous people, and destroy the human species (Schmeltzer 2019) have turned into conspiracy theories. Accountability is crucial in integrating AI systems into society (Shepherd and Majchrzak 2022; Weber et al. 2024. Because AI and decision‐making processes can create a highly intertwined relationship between humans and machines (e.g., AI), decision‐makers or managers may avoid moral responsibility by attributing negative situations to AI (Meissner and Narita, 2023).

Therefore, it has become necessary to comprehensively consider AI's ethical dimensions (Teo et al. 2023). When studies on the use and development of AI technology are considered, ethical issues arise concerning privacy, bias, transparency, and responsibility (Akinrinola et al. 2024; Huriye 2023). There are societal prejudices about AI applications and algorithms, as well as concerns about the violation of the principle of privacy regarding personal data. Moreover, the compliance of developers and administrators with the principles of transparency and responsibility can be seen as factors that significantly affect the acceptance of AI in society (Yazdani and Darbani 2023). Problems concerning implementing ethical principles can result in AI conspiracy theories (Akhter et al. 2024; Liu et al. 2023).

In addition, many conspiracy theories popularized during COVID‐19 are thought to have contributed to the spread of conspiracy theories in different fields (Douglas 2021; Stein et al. 2021; Stojanov and Hannawa 2023). Moreover, if individuals believe in one conspiracy theory, they are more likely to believe in other conspiracy theories (Freeman et al. 2022; Miller 2020). For example, as beliefs in COVID‐19 conspiracy theories increased, beliefs in COVID‐19 vaccination conspiracy theories also increased (Gökalp et al. 2025). This is because conspiracy theories are predicted by a frame of mind that tends to believe in conspiracy theories (Uscinski and Parent 2014).

It has also been reported that individuals who are predisposed to believe in conspiracy theories pay less attention to external sources of information (i.e., scientists, official sources) that are accepted as reliable by the general public, ignore sources that provide information that contradicts their personal beliefs, and tend to believe more speculative sources (Freeman et al. 2022; Imhoff et al. 2022; Rosman et al. 2021). Conspiracy theories are based on low‐quality and weak evidence with gaps and vague details (Brotherton et al. 2013). Individuals who are firmly committed to epistemic rationality and who think analytically are less likely to believe in conspiracy theories (Ståhl and Van Prooijen 2018).

In contemporary society, where fake news and alternative facts are popular with the influence of social media (e.g., Brailovskaia et al. 2021), conspiracy beliefs about AI (a relatively new phenomenon) and its function in future societies have a high potential to emerge. To show how and in what way conspiracy beliefs towards AI develop and to conduct new studies that can provide novel perspectives on this topic, there is a need for a psychometric instrument that helps to define the conceptual framework of AI conspiracy beliefs. Identifying individuals who are prone to conspiracy theories can provide insights into preventing psychological conditions that negatively affect individuals' mental health, such as social isolation, anxiety, PTSD, and paranoid thoughts (Martinez et al. 2022). Moreover, it is also important to determine which emotional needs individuals act on in the context of conspiracy beliefs (Wheeler 2021). Therefore, the first step in developing effective ways to combat social negativities such as resistance to change, health security, misinformation, polarization, narcissism, and insecurity associated with conspiracy beliefs may be to identify individuals who are prone to conspiracy beliefs (Enders et al. 2023). It is important to take preventive measures such as training, media literacy, and activities to prevent these negativities and to develop critical thinking. This situation is also important regarding establishing public health and creating an environment of trust.

The present study aimed to develop a new instrument assessing AI conspiracy beliefs (i.e., the Artificial Intelligence Conspiracy Beliefs Scale [AICBS]) and to examine its psychometric properties. To date, available literature has provided instruments for the assessment of general conspiracy beliefs (Brotherton et al. 2013; Bruder et al. 2013; Stojanov and Halberstadt 2019; Stojanov and Hannawa 2023), AI anxiety (Wang and Wang 2019), and fear of AI (Kieslich et al. 2021). However, an instrument for assessing AI conspiracy beliefs is lacking. Therefore, the development of the AICBS addresses an important research gap and provides a novel instrument for future studies to assess AI conspiracy beliefs. In addition, the spread of AI conspiracy beliefs may cause fear and prejudice towards AI at the societal level. As a result, there may be resistance against the integration of AI research and applications. For this reason, the AICBS can help to understand the reasons behind the conspiracy beliefs towards AI, to reveal their foundations, to determine their limits, and to predict the risks.

## 2 Method

### 2.1 Participants and Recruitment Procedure

A convenience sampling technique was employed to select participants from various parts of Türkiye. The data collection process was conducted online using *Google Forms*. The survey link was advertised on internet forums and social networking sites such as *Facebook* and *WhatsApp* in Türkiye. All participants had to be 18 years or older and provide their written informed consent before starting the online survey anonymously. There were no missing data because the survey could not be submitted unless all questions were answered. No incentive was given to the participants. Data were collected between May and June 2024. Table 1 provides information about the participants (*N *= 788).

**TABLE 1:** Descriptive statistics of the demographic variables.

		Mean (SD) or *n* (%)	
	Entire sample (*N* = 788)	EFA subsample (*n* = 423)	CFA subsample (*n* = 365)
Age	25.10 (8.27)	24.72 (8.10)	25.55 (8.45)
Gender			
Female	442 (56%)	235 (56%)	207 (57%)
Male	346 (44%)	188 (44%)	158 (43%)
Educational level			
High school	92 (11.7%)	58 (13.7%)	34 (9.3%)
Undergraduate	407 (51.6%)	217 (51.3%)	190 (52.1%)
Graduate	289 (36.7%)	148 (35.0%)	141 (38.6%)

Abbreviations: CFA, confirmatory factor analysis; EFA, exploratory factor analysis.

Most participants were female (subsample 1: 56%, subsample 2: 57%, entire sample: 56%). Participants were generally young adults (entire sample *M*_age _= 25.10 years, SD = 8.27). More than half of the participants were university students (51.6%). In addition, the participant's average daily social media use time was 3.65 hours (SD = 2.01).

### 2.2 Measure Development

Before commencing data collection, ethics approval was obtained from the first author's university ethics committee. The AICBS was developed based on principles proposed by DeVellis and Thorpe (2022). Because a comprehensive literature review indicated there were no existing scales assessing AI conspiracy beliefs, conspiracy theories about AI in social networks and studies examining AI concerns were examined (Wang and Wang 2019; Zou and Liu 2023). Following this, an item pool comprising 49 items (e.g., *AI systems will surpass human intelligence and eventually become capable of ruling humans*) with five subdimensions (Global Control [GC], Disinformation [DIS], Human Labor and Human Intelligence [HUM], Arms Rivalry and World Peace [ARM], and Interpersonal Relationships and Social Influence [INT]) was generated (see Table A1 in Supporting Information).

The increase in the scores obtained from the 5‐point Likert‐type scale (1 = *Strongly disagree*, 5 = *Strongly agree*) was interpreted as an increase in the conspiracy belief levels of individuals towards AI. The AICBS was developed in the Turkish language and named the AICBS. Three Turkish language experts and four measurement and evaluation experts evaluated the 49 items regarding content validity, grammar, and semantic clarity. In line with the experts’ opinions, some items were revised, and five items were removed from the item pool. For example, the item *The power behind the world order controlled by AI will be only a handful of people and will direct humanity in line with their own interests* was revised in line with expert opinion to *Only a handful of humans will be the power behind an AI‐controlled world order*. After deleting items with a low factor loading (i.e., < 0.4) or having a cross‐loading problem (i.e., > 0.4 in two or more factors; please see the *Data analysis* section for details), the final AICBS comprised 30 items and five subdimensions: GC: 8 items; DIS: 5 items; HUM: 7 items; ARM: 5 items; and INT: 5 items.

### 2.3 Other Measures

*Generic Conspiracist Beliefs Scale (GCB‐5)*. The five‐item GCB‐5 (Kay and Slovic 2023) was used to assess conspiracist beliefs. The items (e.g., *Evidence of alien contact is being concealed from the public*) are rated on a 6‐point Likert‐type scale (1 = *strongly disagree*; 6 = *strongly agree*). Higher scores indicate greater conspiracist beliefs. Previous studies have indicated the validity and reliability of the GCB‐5 (Dagnall et al. 2023; Liekefett et al. 2024). Because the GCB‐5 has never been translated and validated into Turkish, the present study used the standard translation procedure (i.e., forward translation, back translation, and reconciliation) to translate the GCB‐5 into the Turkish language. In the present study, the psychometric properties of the GCB‐5 were good (see Table A3 in Supporting Information).

*Anomie Scale (AS)*. The three‐item AS (Goertzel 1994) was used to assess anomie. Items (e.g., *I think that the life of an ordinary person is getting worse every day*) are rated on a 5‐point Likert‐type scale (1 = *strongly disagree*; 5 = *strongly agree*). Higher scores indicate greater levels of anomie. Because the three‐item AS has never been translated and validated into Turkish, a standard translation procedure (i.e., forward translation, back translation, and reconciliation) was used to translate the AS into the Turkish language. The psychometric properties were adequate in the present study (Cronbach's *α* and McDonald's *ω* were 0.651 and 0.654, respectively).

*Artificial Intelligence Anxiety Scale (AIAS)*. The Turkish 21‐item AIAS (Terzi 2020; Wang and Wang 2019) was used to assess AI anxiety. The AIAS has four subdimensions (learning, job replacement, social blindness, and AI configuration). Items (e.g., *I don't know why, but humanoid AI techniques/products (e.g., humanoid robots) scare me*) are rated on a 7‐point scale (1 = *strongly disagree*; 7 = *totally agree*). Higher scores indicate greater AI anxiety. The learning subdimension was unrelated to AI conspiracy theories, so it was not used in the present study. In the present study, the internal consistencies of the AIAS subdimensions were very good (job replacement: *α* = 0.881; *ω* = 0.885; social blindness: *α* = 0.826; *ω* = 0.832; AI configuration: *α* = 0.906; *ω* = 0.906).

### 2.4 Data Analysis

The study sample was randomly divided into two subsamples: (i) an exploratory factor analysis (EFA) subsample to explore the initial factor structure of the AICBS and (ii) a confirmatory factor analysis (CFA) subsample to verify the factor structure derived from the EFA. The second subsample was then used to examine the AICBS's discriminant validity based on the heterotrait–monotrait (HTMT) ratio method. The two subsamples were sufficient for each factor analysis according to the recommended minimum item–participant ratio of 5 to 1 (i.e., each item needs a minimum of five participants) (Lorenzo‐Seva and Ferrando 2024). Given that the original version of AICBS contained 44 items, 220 participants for each factor analysis were deemed to be sufficient. In addition, the entire sample was used for the following analyses: internal consistency, concurrent validity with external criterion measures, and difference tests between gender and educational levels. The CFA, HTMT ratio, and internal consistency analyses were performed using JASP 0.18.3; the rest was performed using IBM SPSS version 25.0.

The EFA was performed using the following steps: (i) checking if the subsample was adequate for factor analysis via the Kaiser–Mayor–Olkin (KMO) test (i.e., to check if the items in the AICBS had sufficient explained variance to extract factors), where a KMO value > 0.7 indicates adequacy for factor analysis (Field 2024); (ii) using principal axis factoring extraction to extract the factors using the Kaiser rule (i.e., number of extracted factors is based on how many factors have an eigenvalue > 1) (Ledesma and Valero‐Mora 2007); (iii) adopting the promax oblique rotation method to identify the item‐factor relationship; (iv) examining the factor loading for every item by removing any item having a loading < 0.4 or any item having cross‐loading (i.e., one item has two or more loadings > 0.4) (Field 2024); and (v) repeating steps (iii) and (iv) until all items have only one‐factor loading > 0.4 in a factor. A short five‐item version of the AICBS (i.e., AICBS‐5) was also developed using the EFA. Specifically, the item in each AICBS subdimension with the highest loading was used to generate the AICBS‐5. The AICBS‐5 was also tested using EFA to examine if it could simplify the multidimensional AICBS into a unidimensional measure.

After using EFA to derive the factor structure of the AICBS, the entire AICBS with all its factors and the AICBS‐5 were examined for their internal consistency using both Cronbach's *α* and McDonald's *ω*. A value > 0.7 in Cronbach's *α* or McDonald's *ω* indicates good internal consistency (George and Mallery 2016). Then, CFA was performed using the maximum likelihood estimator for both the AICBS and AICBS‐5. The following fit indices calculated from the CFA were used to define if the factor structure derived from the prior EFA results was verified: comparative fit index (CFI) > 0.9, Tucker–Lewis index (TLI) > 0.9, root mean square error of approximation (RMSEA) < 0.08, and standardized root mean square residual (SRMR) < 0.08 (Lin et al. 2018; Whittaker and Schumacker 2022). The factor loadings derived from the AICBS CFA were then used for the HTMT method, and discriminant validity is supported when an HTMT ratio is lower than 0.85 (Kline 2023).

The entire AICBS with all its factors and the AICBS‐5 were examined for concurrent validity with relevant measures (i.e., the external criterion measures of AIAS, AS, and GCB‐5). Pearson correlations were used for the concurrent validity, and coefficients > 0.3 indicated moderate or stronger correlations (Cohen 1988). Lastly, the entire AICBS with its factors and the AICBS‐5 were examined to see if their scores were significantly different in gender groups (i.e., male vs. female) and educational level groups (i.e., high school, undergraduate, and graduate). An independent *t*‐test was used to compare genders; analysis of variance (ANOVA) with Bonferroni adjustment was used for comparison between educational levels. More specifically, the adjusted alpha level was set at *p *< 0.016 to indicate significance.

## 3 Results

Table 2 shows the final factor structure and factor loadings based on EFA for the AICBS. The KMO value was acceptable for conducting EFA, and EFA results suggested a five‐factor structure for the AICBS. All items loaded on the expected five factors (62.58% of total variance explained), although some items were deleted due to low factor loadings (see Table 2). The EFA results supported the unidimensional structure of the AICBS‐5. The factor loadings of the AICBS‐5 items ranged between 0.590 and 0.740 (see Table 2).

**TABLE 2:** The final factor structure and factor loadings of the Artificial Intelligence Conspiracy Beliefs Scale based on exploratory factor analysis.

	GC	DIS	HUM	ARM	INT
GC1	0.524				
GC2	0.790				
GC3	0.786				
GC5	0.882				
GC6	0.746				
GC7	0.678				
GC9	0.814				
GC12	0.537				
DIS1		0.419			
DIS2		0.482			
DIS3		0.616			
DIS4		0.756			
DIS5		0.679			
HUM1			0.665		
HUM2			0.422		
HUM4			0.552		
HUM5			0.656		
HUM6			0.844		
HUM7			0.646		
HUM8			0.594		
ARM2				0.533	
ARM3				0.632	
ARM4				0.705	
ARM5				0.650	
ARM6				0.688	
INT3					0.605
INT4					0.719
INT5					0.520
INT6					0.608
INT7					0.640

*Note*: The item numbers were reported based on the original item number without item deletion. The extraction method was principal axis factoring, and the rotation method was promax oblique rotation. Factor loading values < 0.4 are not reported.

Abbreviations: ARM, Arms Rivalry and Less World Peace; DIS, Disinformation; GC, Global Control; HUM, Human Labor and Human Intelligence; INT, interpersonal Relationships and Social Influence.

Table 3 shows the CFA results for the AICBS. The five‐factor structure of the AICBS found in the EFA was confirmed by the acceptable fit of the CFA fit indices of the second subsample (i.e., CFI = 0.913; TLI = 0.904; RMSEA = 0.064; and SRMR = 0.049). Because the HTMT ratio of factor loadings was less than 0.85, discriminant validity was supported. CFA results confirmed the unidimensional structure of AICBS‐5 obtained with EFA. CFA results for AICBS‐5 indicated a significant and acceptable fit (i.e., CFI = 0.988; TLI = 0.976; RMSEA = 0.057; and SRMR = 0.020). The entire AICBS, its subdimensions, and AICBS‐5 had reliable internal consistency coefficients (see Table 3).

**TABLE 3:** Scale properties of the Artificial Intelligence Conspiracy Beliefs Scale (AICBS).

	AICBS	GC	DIS	HUM	ARM	INT	AICBS‐5
Cronbach's *α* ^a^	0.954	0.898	0.811	0.885	0.873	0.873	0.799
McDonald's *ω* ^a^	0.955	0.899	0.818	0.888	0.875	0.873	0.804
EFA^b^							
Eigenvalue	—	12.58	2.32	1.57	1.20	1.11	—
Variance explained	—	41.94	7.73	5.22	4.00	3.69	45.28
KMO	0.947	—	—	—	—	—	0.826
CFA^c^							
χ^2^ (df)	979.80 (393)	—	—	—	—	—	11.1 (5)
*p* value	< 0.001	—	—	—	—	—	< 0.05
CFI	0.913	—	—	—	—	—	0.988
TLI	0.904	—	—	—	—	—	0.976
RMSEA	0.064	—	—	—	—	—	0.057
SRMR	0.049	—	—	—	—	—	0.020
HTMT method^c^							
GC	—	1.00					—
DIS	—	0.73	1.00				—
HUM	—	0.75	0.79	1.00			—
ARM	—	0.63	0.67	0.80	1.00		—
INT	—	0.66	0.76	0.84	0.75	1.00	—

Abbreviations: ARM, Arms Rivalry and Less World Peace; CFA, confirmatory factor analysis; CFI, comparative fit index; DIS, Disinformation; EFA, exploratory factor analysis; GC, Global Control; HTMT, heterotrait–monotrait ratio; HUM, Human Labor and Human Intelligence; IFI, incremental fit index; INT, Interpersonal Relationships and Social Influence; KMO, Kaiser–Meyer–Olkin measure of sampling adequacy; RMSEA, root mean square error of approximation; SRMR, standardized root mean square residual; TLI, Tucker–Lewis index.

^a^ Based on the entire sample.

^b^ Based on the EFA subsample.

^c^ Based on CFA subsample.

Table 4 shows correlations between the whole AICBS, its subdimensions, the AIAS subdimensions, AS, GCB‐5, and AICBS‐5. The AICBS had a strong positive correlation with all subdimensions of AIAS and GCB‐5 (*r* ≥ 0.49) and a moderate positive correlation with the AS (*r* = 0.30–0.49). AICBS‐5 was strongly positively correlated with AIAS subdimensions and GCB‐5, and moderately positively correlated with the AS.

**TABLE 4:** Concurrent validity of the Artificial Intelligence Conspiracy Beliefs Scale (AICBS).

	Pearson correlation with an external criterion measure				
	AIAS: Job Replacement	AIAS: Social Blindness	AIAS: AI Configuration	Anomie Scale	GCB‐5
AICBS	0.65	0.62	0.56	0.38	0.51
GC	0.52	0.50	0.48	0.28	0.44
DIS	0.46	0.47	0.41	0.30	0.42
HUM	0.62	0.56	0.54	0.36	0.43
ARM	0.56	0.56	0.47	0.33	0.46
INT	0.56	0.52	0.43	0.34	0.38
AICBS‐5	0.60	0.57	0.53	0.35	0.45

*Note*: All *p* values < 0.01. AICBS‐5 is a five‐item short version of the AICBS.

Abbreviations: AIAS, Artificial Intelligence Anxiety Scale; ARM, Arms Rivalry and World Peace; DIS, Disinformation; GC, Global Control; GCB‐5, Generic Conspiracist Beliefs Scale‐5; HUM, Human Labor and Human Intelligence; INT, Interpersonal Relationships and Social Influence.

Table 5 shows the differentiation of AICBS, its subdimensions, and AICBS‐5 according to gender and education level. The mean scores obtained from the AICBS, its subdimensions, and the AICBS‐5 differed statistically significantly between genders. Females had higher scores than males (see Table 5).

**TABLE 5:** Comparing the Artificial Intelligence Conspiracy Beliefs Scale (AICBS) between gender and educational level.

	Mean (SD) in gender			Mean (SD) in educational level			
	Female	Male	*t* (*p*)	High school	Undergraduate	Graduated	*F* (*p*)
AICBS	3.73 (0.67)	3.56 (0.72)	3.44 (< 0.001)	3.51 (0.71)	3.63 (0.68)	3.75 (0.70)	5.10 (0.01)^a^
GC	3.54 (0.88)	3.30 (0.93)	3.65 (< 0.001)	3.29 (0.94)	3.41 (0.86)	3.51 (0.96)	2.32 (0.10)
DIS	3.74 (0.78)	3.58 (0.83)	2.76 (0.01)	3.48 (0.88)	3.62 (0.76)	3.79 (0.81)	6.73 (< 0.001)^a^, ^b^
HUM	3.99 (0.82)	3.82 (0.89)	2.79 (0.01)	3.78 (0.86)	3.88 (0.87)	4.01 (0.83)	3.23 (0.04)
ARM	4.04 (0.83)	3.92 (0.91)	2.02 (0.04)	3.88 (0.86)	3.96 (0.87)	4.06 (0.86)	2.26 (0.11)
INT	4.08 (0.81)	3.91 (0.87)	2.86 (< 0.001)	3.80 (0.85)	3.98 (0.84)	4.11 (0.83)	5.26 (0.01)^a^
AICBS‐5	3.97 (0.73)	3.74 (0.81)	4.24 (< 0.001)	3.75 (0.81)	3.86 (0.76)	3.92 (0.77)	1.706 (0.18)

*Note*: AICBS‐5 is a five‐item short version of the AICBS.

Abbreviations: ARM, Arms Rivalry and World Peace; DIS, Disinformation; GC, Global Control; HUM, Human Labor and Human Intelligence; INT, Interpersonal Relationships and Social Influence.

^a^ Indicates a significant difference between high school and graduate participants.

^b^ Indicates a significant difference between undergraduate and graduate participants using Bonferroni adjustment.

The mean scores of GC, HUM, ARM subdimensions, and AICBS‐5 did not differ statistically significantly according to the gender groups. This means that gender does not have a statistically significant effect on the aforementioned variables. AICBS, DIS, and INT subdimension mean scores differed significantly according to educational level. Following Bonferroni correction, the findings indicated that graduates had higher scores than individuals with high school as the highest education level on the AICBS (*M* = 3.75 > *M* = 3.51), DIS (*M* = 3.79 > *M* = 3.48), and INT (*M* = 4.11 > *M* = 3.80). There was also a significant difference in the DIS subdimension, with graduates scoring higher than undergraduates (*M* = 3.79 > *M* = 3.62).

## 4 Discussion

In recent years, the rapid development of AI has caused uncertainty and unpredictability about what this technology may cause in the future (Nan et al. 2023). Therefore, it is important to assess conspiracy beliefs regarding the future state of AI. Identifying individuals with conspiracy beliefs provides insight into their psychological conditions and the emotional needs that drive their actions (Douglas and Sutton 2023; Marchlewska et al. 2022). Therefore, it can provide an important basis for developing an effective defense mechanism against the spread of false information and the resulting environment of insecurity. In this context, education and awareness‐raising activities can direct individuals to more robust sources of information and strengthen their critical thinking skills (Georgiu et al. 2021). Consequently, individuals may become able to recognize the implausible aspects of conspiracy theories more easily.

The AICBS was developed to understand the reasons underlying AI conspiracy beliefs. Predicting possible risks may have the potential to make significant contributions to the gap in the literature. The five‐factor structure of the AICBS explained 62.58% of the total variance with good psychometric properties, demonstrating good internal and external validity as well as very good internal consistency. Moreover, the short unidimensional version of the scale (AICBS‐5) was additionally developed and also showed very good internal consistency. Based on this psychometric evaluation, both the AICBS (with a five‐factor structure) and the AICBS‐5 (with a unidimensional structure) are valid instruments for assessing AI conspiracy beliefs.

Conspiracy theories are usually based on the belief that a secret and harmful plan is being carried out (Hodapp and Von Kannon 2008). Therefore, these beliefs are unlikely to be based on empirical evidence. The development of such a psychometric measurement tool specifically on AI conspiracies could have impacts regarding the motivations underlying conspiracy theories and contribute to a deeper and more nuanced understanding of their role. The AICBS and AICBS‐5 have the potential to help researchers to understand how AI conspiracy theories work and how they evolve in the context of anxiety, worry, and fear.

Using the AICBS or AICBS‐5, relevant stakeholders (e.g., government personnel) can identify individuals’ conspiracy theory tendencies and design appropriate programs to prevent their spread. Consequently, it may be possible to reduce the potential negative impact on societal acceptance and integration of AI and to understand the structural characteristics and prevalence of AI‐related conspiracy theories. In addition, the AICBS can help develop specific intervention strategies for the individual, society, and institutions to reduce misinformation about AI and create a healthy, evidence‐based perspective. Therefore, the AICBS can be used in needs analysis to develop training and awareness‐raising programs.

The question of whether AI is a threat or an opportunity for humanity shows the complexity and unpredictability of the relationship between humanity and AI (Zimmerman et al. 2024). Accordingly, this relationship will have ethical and social consequences. In terms of ethics, the question of who is responsible for AI's actions creates serious concerns (Huriye 2023; Pflanzer et al. 2023). Future empirical studies using the AICBS or AICBS‐5 may help to show concrete indicators of these concerns.

It is expected that with the emergence of AGI and ASI types of AI, questions about the future of humanity and the meaning of being human will be raised (Kelly et al. 2023; Putnik et al. 2021). Discussions on these questions are likely to lead to the spread of AI conspiracy theories to the masses. From this point of view, the AICBS can contribute to understanding the reception of AI conspiracy beliefs and help take measures against possible adverse reactions. In addition, the spread of AI conspiracy beliefs may cause fear and prejudice towards AI at the societal level. As a result, there may be resistance against the integration of AI research and applications.

### 4.1 Limitations and Directions for Future Research

Although the AICBS and its short form AICBS‐5 were developed as a consequence of rigorous and detailed methods, they have some limitations. First, test–retest reliability was not assessed for either the AICBS or the AICBS‐5. Therefore, it is unclear whether both measures are valid to be used in studies with several measurement time points. Second, due to the convenience sampling method used for data collection, the generalizability of the present findings is restricted. Future research should use more representative sampling techniques to test the AICBS and AICBS‐5. Third, the responses to the AICBS and AICBS‐5 relied on self‐report. Therefore, participants may not have disclosed their true feelings due to factors such as social desirability. AI conspiracy beliefs can be examined holistically in studies where different types of measurement tools are used together. For example, the AI Self‐Efficacy Scale (AISES; Wang and Chuang 2024), developed to assess the AI self‐efficacy levels of educators and practitioners, could be included in the same study as the AICBS or the AICBS‐5. Therefore, it can be tested whether AI conspiracy beliefs have an effect on AI self‐efficacy behavior. Fourth, the AICBS was developed in the context of the Turkish culture. The psychometric structure of the AICBS needs to be evaluated in different cultures and languages. Future studies are needed to both replicate and validate the scale in other languages to ensure that the AICBS is both reliable and valid worldwide.

## 5 Conclusions

The AICBS is a useful psychometric instrument for understanding AI conspiracy beliefs. The AICBS and its short form (AICBS‐5) have good psychometric properties, including internal consistency and concurrent validity. They can be considered valid and reliable psychometric instruments for the assessment of AI conspiracy beliefs. In large‐scale studies with many variables, the AICBS‐5 can help researchers to test complex mechanisms involving AI conspiracy beliefs and to reduce survey fatigue.

## Author Contributions

**Chung‐Ying Lin**: conceptualization, investigation, writing – original draft, writing – review and editing, visualization, validation, methodology, software, formal analysis, project administration, resources, supervision, data curation. **Julia Brailovskaia**: supervision, writing – original draft, writing – review and editing, methodology, project administration. **Servet Üztemur**: conceptualization, investigation, funding acquisition, writing – original draft, writing – review and editing, visualization, validation, methodology, software, formal analysis, project administration, resources, supervision, data curation. **Ali Gökalp**: Methodology, validation, visualization, writing – review and editing, writing – original draft, investigation, conceptualization, software, formal analysis, resources, data curation. **Nail Değirmenci**: conceptualization, methodology, software, data curation, formal analysis, investigation, visualization. **Po‐Ching Huang**: conceptualization, investigation, writing – original draft, writing – review and editing, visualization, validation, methodology, software, formal analysis, data curation. **Hua Chen**: investigation, writing – original draft, writing – review and editing, methodology, visualization. **Mark D. Griffiths**: writing – review and editing, writing – original draft, project administration, supervision, methodology. **Amir H. Pakpour**: methodology, writing – review and editing, validation, investigation, software, formal analysis.

## Ethics Statement

All procedures performed in studies involving human participants were in accordance with the ethical standards of the institutional and/or national research committee and with the 1964 Helsinki Declaration and its later amendments or comparable ethical standards. The Anadolu University Ethics Committee (Ethics Number: 732773) granted ethical approval.

## Informed Consent

Informed consent was obtained from all participants included in the study.

## Conflicts of Interest

The authors declare no conflicts of interest.

## Peer Review

The peer review history for this article is available at https://publons.com/publon/10.1002/brb3.70648

## Supporting information




**Supplementary Tables**: brb370648‐sup‐0001‐Appendix.docx

## Data Availability

The original form and the study's data are available from the co‐corresponding authors upon reasonable request.
