# Supplementary material for: Dark Future: Development and Initial Validation of Artificial Intelligence Conspiracy Beliefs Scale (AICBS)
Source: Brain Behav. 2025 Jul 3;15(7):e70648. doi: 10.1002/brb3.70648 (PMC12224044; doi:10.1002/brb3.70648)
Supplement: Supplementary file 1 — Supplementary Tables: brb370648‐sup‐0001‐Appendix.docx [file BRB3-15-e70648-s001.docx]

**Table 1.** Artificial Intelligence Conspiracy Beliefs Scale

| ***Global Control*** |
| --- |
| 1. Only a handful of people will be the power behind the world order controlled by AI. |
| 1. AI systems will surpass human intelligence and eventually become capable of ruling humans. |
| 1. AI will use the internet to control people. |
| 1. Human nature will not only be copied, but original humanoid robot species will emerge. |
| 1. By gaining consciousness and self-awareness, AI will get out of control and start a war against humanity. |
| 1. AI will take over all electronic systems, from smartphones to televisions, and make humans its prisoners. |
| 1. AI will decide who will survive by manipulating personal data. |
| 1. Some secret groups or organizations will use AI technologies to manipulate and control people. |
| 1. AI will take over humanity and commit massacres around the world. |
| 1. AI will violate human rights because it lacks emotion. |
| 1. With AI, human life will become much less valuable than it already is. |
| 1. AI will be used to control people's minds and direct them as they wish. |
| ***Disinformation*** |
| 1. AI will make people not access accurate information. |
| 1. AI decision systems will increase racism worldwide. |
| 1. AI will become a key tool used to manipulate voters through fake news. |
| 1. Users will be skeptical of all kinds of information and news on social media due to AI-generated disinformation. |
| 1. AI-generated disinformation will lead to chaos. |
| ***Human Labor and Human Intelligence*** |
| 1. People will unwittingly fall under the influence of AI and lose their freedom. |
| 1. People will be disconnected from the real world due to AI-supported virtual assistants. |
| 1. Due to job losses caused by AI, capital and profit will be shared among a much smaller group of people and inequalities will increase. |
| 1. AI will replace teachers and educate students according to the ideology of the dominant power. |
| 1. AI will accustom people to laziness, resulting in dumb generations. |
| 1. AI will destroy human creativity skills (painting, sculpture, music, etc.). |
| 1. Due to AI, people will find it very difficult to distinguish between reality and imagination. |
| 1. AI will interfere with people's subconscious and people will be captive to AI without realizing it. |
| 1. AI will drive people into poverty by destroying many professions. |
| 1. People will be unemployed due to the widespread use of humanoid robots. |
| 1. Privacy will lose its importance as all kinds of private information will be accessed through AI. |
| 1. Personal data will be captured by global companies using AI technology. |
| ***Arms Competition and Less World Peace*** |
| 1. The malicious use of AI in security systems will lead to irreparable disasters. |
| 1. The use of AI in military applications will endanger national security. |
| 1. AI applications misused by malicious people will cause war between states. |
| 1. The use of AI in critical infrastructure systems will endanger the security of many military facilities. |
| 1. Due to AI, super-lethal weapons will be developed and the destructive impact of wars will increase. |
| 1. AI will cause wars between countries by remotely controlling and firing nuclear weapons. |
| 1. Cyber wars will increase rapidly with AI. |
| ***Interpersonal Relationships and Social Impact*** |
| 1. AI will make social media more attractive, leading to high social media addiction levels in individuals. |
| 1. Personalized content through AI will destroy the value of respect for differences. |
| 1. Personalized content through AI will prevent intellectual diversity. |
| 1. Personalized content through AI will lead to less interaction and increase social isolation. |
| 1. Personalized content through AI will remove the ability to empathize. |
| 1. Personalized content through AI will cause individuals to disconnect from their social interactions. |
| 1. Big companies will change people's behavior through smart machines using AI. |
| 1. The use of AI-based technologies will cause people to become dependent on AI for many things, and as a result, they will lose their ability to think and reason. |

**Table 2.** Artificial Intelligence Conspiracy Beliefs Scale**-**5 (AICBS-5)

| 1. AI will take over humanity and commit massacres around the world. |
| --- |
| 2. AI-generated disinformation will lead to chaos. |
| 3. AI will accustom people to laziness, resulting in dumb generations. |
| 4. AI will cause wars between countries by remotely controlling and firing nuclear weapons. |
| 5. Big companies will change people's behavior through smart machines using AI. |

**Table 3.** Generic Conspiracist Beliefs Scale-5 and Anomie Scale

| **Generic conspiracist beliefs Scale – 5* (Kay & Slovic, 2023)** | 1. The government permits or perpetrates acts of terrorism on its own soil, disguising its involvement. |
| --- | --- |
|  | 2. Evidence of alien contact is being concealed from the public. |
|  | 3. New and advanced technology which would harm current industry is being suppressed. |
|  | 4. Certain significant events have been the result of the activity of a small group who secretly manipulate world events. |
|  | 5. Experiments involving new drugs or technologies are routinely carried out on the public without their knowledge or consent. |
| **Anomie (Goerzel, 1994)** | 1. I think the life of an ordinary man is getting worse day by day |
|  | 2. It is hardly fair to bring a child into today's world |
|  | 3. I think public officials do not care about the problems of the common man |

*According to the results of the CFA conducted to adapt the GCB-5 to Turkish culture, it was found that the scale preserved its original structure and the fit indices were at an excellent level: (χ^2^ [df] = 0.076 [1]; CFI = 1.00; TLI = 1.00; RMSEA = 0.000; SRMR = 0.001; *p*>.05). The Cronbach's α and McDonald's ω values of the Turkish version of the scale were 0.70 and 0.70, respectively.

**Table 5.** Item properties of the Artificial Intelligence Conspiracy Beliefs Scale

|  | **Ceiling effect (%)** | **Floor effect (%)** | **Skewness** | **Kurtosis** | **Mean (SD)** |
| --- | --- | --- | --- | --- | --- |
| **Global Control** | **7.4** | **.08** | **-.414** | **-.528** | **3.55 (.95)** |
| GC1 | 21.1 | 6 | -.632 | -.537 | 3.57 (1.12) |
| GC2 | 23.06 | 5.8 | -.537 | -.842 | 3.54 (1.21) |
| GC3 | 22.02 | 5.5 | -.447 | -.785 | 3.50 (1.17) |
| GC5 | 21.06 | 6.8 | -.205 | -1.055 | 3.34 (1.23) |
| GC6 | 30.07 | 6.6 | -.598 | -.759 | 3.63 (1.24) |
| GC7 | 17.03 | 10.07 | -.054 | -1.109 | 3.13 (1.26) |
| GC9 | 16.04 | 9.06 | -.167 | -.892 | 3.20 (1.21) |
| GC12 | 29.03 | 4.07 | -.661 | -.476 | 3.70 (1.15) |
| **Disinformation** | **11.2** | **1.1** | **-.133** | **-.458** | **3.48 (.92)** |
| DIS1 | 17.03 | 8.02 | 1.21 | -.983 | 3.18 (1.21) |
| DIS2 | 17.5 | 6 | -.258 | -.753 | 3.35 (1.14) |
| DIS3 | 32.6 | 3 | -.887 | .318 | 3.90 (1.03) |
| DIS4 | 38.9 | 2.7 | -1.046 | .673 | 4.03 (1.01) |
| DIS5 | 39.7 | 1.6 | -1.010 | .591 | 4.07 (.96) |
| **Human Labour and Human Intelligence** | **34.8** | **1.1** | **-1.187** | **1.154** | **4.16 (.89)** |
| HUM1 | 46 | 1.9 | -.998 | .120 | 4.08 (1.06) |
| HUM2 | 50.7 | 2.7 | -1.404 | 1.444 | 4.21 (1.02) |
| HUM4 | 30.1 | 7.7 | -.563 | -.753 | 3.60 (1.25) |
| HUM5 | 52.3 | 2.7 | -1.385 | 1.330 | 4.22 (1.02) |
| HUM6 | 39.2 | 5.2 | -.713 | -.728 | 3.78 (1.26) |
| HUM7 | 41.6 | 3.3 | -.989 | .129 | 3.99 (1.15) |
| HUM8 | 42.7 | 3.8 | -.903 | -.144 | 3.95 (1.15) |
| **Arms Rivalry and Less World Peace** | **25.8** | **.05** | **-1.155** | **.762** | **4.02 (.89)** |
| ARM2 | 40.8 | 3.3 | -.883 | -.040 | 3.95 (1.10) |
| ARM3 | 41.6 | 2.5 | -1.042 | .511 | 4.05 (1.02) |
| ARM4 | 38.1 | 3.6 | -.949 | .316 | 3.96 (1.07) |
| ARM5 | 47.9 | 2.5 | -1.155 | .762 | 4.14 (1.02) |
| ARM6 | 42.5 | 4.4 | -1.017 | .279 | 3.98 (1.12) |
| **Interpersonal Relationships and Social Influence** | **23.3** | **.03** | **-.817** | **.341** | **4.03 (.85)** |
| INT3 | 39.7 | 2.7 | -.891 | .052 | 3.97 (1.07) |
| INT4 | 40 | 1.4 | -.886 | .044 | 4.03 (1.01) |
| INT5 | 41.1 | 2.7 | -1.008 | .390 | 4.03 (1.04) |
| INT6 | 41.9 | 1.4 | -.949 | .150 | 4.06 (1.01) |
| INT7 | 42.2 | 2.7 | -1.119 | .905 | 4.09 (1) |
